# All roads can lead to surgery

**DOI:** 10.1016/j.amsu.2021.103147

**Published:** 2021-12-09

**Authors:** D. Motter, F. Salimi

**Affiliations:** Grange University Hospital, United Kingdom

**Keywords:** Surgical training, Surgery, Core competence, UK

## Abstract

Pursuing a career in surgery is a rigorous process that demands commitment and hard work. Surgeons in the United Kingdom must go overcome a myriad of competitive stages prior to becoming a consultant. In the United Kingdom, the most common pathway to become a surgeon is via the ‘direct route’ which encompasses completing multiple training programs, namely, the Foundation Program, followed by Core Surgical Training, and then onto Higher Speciality Training, with the aim of obtaining the Certificate of Completion of Training (CCT). More recently, certain specialities have introduced the ‘Improving Surgical Training’ (IST) pathway. IST is a competence-based, run through surgical program that was introduced by the Royal College of Surgeons of England (RCS) and Health Education England (HEE). “The pilot trials improvements in the quality of training, a better balance between service and training for trainees, and professionalisation of the role of the surgical trainers” [1]. However, there are alternative pathways that have been designed for those who prefer a different route to training or have been unsuccessful during the selection stages. These pathways are not well-advertised, but with the competitive rates now higher than before, it is becoming a more attractive pathway for junior doctors, hence the increasing the demand for clearer instructions on potential routes for aspiring surgeons.

## Highlights

• Core surgical training in the UK has become more competitive over the last few years.
• There are different pathways to becoming a surgeon.
• We look at different routes available to junior trainees.
• We encourage trainees who want flexibility at work to consider the alternative core training pathway.

Pursuing a career in surgery is a rigorous process that demands commitment and hard work. Surgeons in the United Kingdom must go overcome a myriad of competitive stages prior to becoming a consultant. In the United Kingdom, the most common pathway to become a surgeon is via the ‘direct route’ which encompasses completing multiple training programs, namely, the Foundation Program, followed by Core Surgical Training, and then onto Higher Speciality Training, with the aim of obtaining the Certificate of Completion of Training (CCT). More recently, certain specialities have introduced the ‘Improving Surgical Training’ (IST) pathway. IST is a competence-based, run through surgical program that was introduced by the Royal College of Surgeons of England (RCS) and Health Education England (HEE). “The pilot trials improvements in the quality of training, a better balance between service and training for trainees, and professionalisation of the role of the surgical trainers” [1]. However, there are alternative pathways that have been designed for those who prefer a different route to training or have been unsuccessful during the selection stages. These pathways are not well-advertised, but with the competitive rates now higher than before, it is becoming a more attractive pathway for junior doctors, hence the increasing the demand for clearer instructions on potential routes for aspiring surgeons.

Junior doctors who wish to pursue a surgical career have to apply for Core Surgical Training (CST), which is a 2-year program, typically with 4–6 months rotations in different surgical specialities. Junior doctors must start early in their Foundation Program to prepare for the CST interview, and maximize their portfolio to successfully progress. The Core Surgical Training Portfolio scoring system comprises of three stations in the CST interview, which include a portfolio, clinical scenario, and a management/leadership station. There is a 2-stage shortlisting process prior to the CST interviews. The first stage involves self-assessment portfolio score which is submitted via Oriel, the second stage involves consultant review of evidence provided. Each year, there are approximately “500 to 600 core surgical training posts available. These jobs are divided up according to deaneries” [2].

Over the last few years, the competition has increased, and many junior doctors are left without surgical training. According to Health Education England “In 2020, 2322 applicants applied for core surgical training, of those, only 605 were successful” [3]. That leaves a staggering 1717 junior doctors without formal training (ratio 3.84). Compared to the number of applicants in 2019, 1896 applied with only 648 posts available (ratio 2.93). In addition to that, junior doctors have felt the brunt of the effects COVID-19 has had on training. There has been a significant increase in number of applicants, and a decrease in the number of posts available. COVID-19 has forced the Royal College of Surgeons to cancel important surgical courses and examinations in a bid to tackle the pandemic. COVID-19 has also disrupted the recruitment process, and the face-to-face interview segment of the process was removed for the 2020 applicants. This meant that trainees would be ranked based solely on the first stage of the recruitment process, the portfolio section, with the interview stage being disregarded.

The vast majority of junior doctors will opt to take a F3 year to improve their CV and surgical skills, and then re-apply the following year. Keeping in mind that the competition ratio will continue to increase as more previously unsuccessful applicants are expected to apply. Despite these limiting factors, Health Education England have not revised the 18-month limit of experience in surgery and those with more than 18 months of surgical experience (excluding time spent in surgery during foundation program) will not be considered for core surgical training. This leaves a small window of 18 months for junior doctors to improve their portfolio.

There is an alternative pathway to Core Surgical Training. International medical doctors often complete the ‘Certificate of Readiness to Enter Higher Surgical Training’ form, also known as the alternative certificate of core surgical training, thus bypassing Core Surgical Training. This certificate is obtained by doctors who have completed their core surgical training abroad but are currently not in a training program in the UK. The ‘Alternative certificate of core surgical training’ is a form that assesses different aspects of being a competent surgeon. This includes the ability to demonstrate generic core surgical skills as well as professionalism and leadership skills. The form must be completed by a consultant or a specialist in surgical fields that the junior doctor has worked closely with. You must have worked with that said consultant/specialist for at least 3 months duration and have exhibited clinical skills equivalent to those undergoing core training. The form can be filled out by multiple consultants. The person signing the form does not have to be registered with the General Medical Council, but they will need to provide evidence of registration with their respective medical regulation body in English.

Whilst this seems like an easier path to take, there is a strict criterion for completing the form. Failure to meet the criteria can result in the suspension of application. An incomplete certificate may lead to the application being ineligible for current recruitment. Consultants are often reluctant to sign the forms off as it requires assessment of a variety of surgical competencies. Both the applicant and the consultant signing off the form must familiarise themselves with the Intercollegiate Surgical Curriculum program (ISCP) core surgical syllabus. It is also worth mentioning that “applicants using the alternative certificate of core surgical training for entry into a surgical specialty will be awarded a Certificate of Eligibility for Specialist Registration – Combined Programme (CESR – CP), rather than a Certificate of Completion of Training (CCT) upon successful completion of their high specialty training program” [4]. In the UK, there is no difference between CCT and CESR. In Europe however, “CESRs aren't recognised in the same way. Instead, the holder must apply for recognition under what the Directive calls ‘the general system for the recognition of evidence of training’. And this is likely to involve a process of assessment” [5]. This is important to bear in mind if you are looking to move to Europe after training.

As a non-trainee doctor, it will be your responsible to ensure the evidence are presented to the consultant appropriately.

The certificate of Readiness to Enter Higher Surgical Training form is 5 pages long, and whilst all evidence is often difficult to present entirely to one consultant, multiple consultants or senior colleagues (e.g. ST5 or above) can witness and sign off specific competencies. It is worth mentioning that your ‘core training’ will not be regulated by the Royal College of Surgeons, and you don't belong to a deanery that overlooks your progress. You will not be assigned an educational or clinical supervisor, and you will not have formal teaching sessions. It is then entirely your responsible to ensure that you have the same exposure as core surgical trainees, enlist yourself in mandatory courses and be up to date with the knowledge and surgical skills. While this might be difficult to achieve in just 3 months' time, it is still a viable option for those who prefer to stay in a local area, have a better work-life balance or have commitments outside of their career. We strongly advise reading the Certificate of Readiness to Enter Higher Surgical Training thoroughly before making a decision. Try to emulate a curriculum for yourself similar to that offered to core surgical trainees. Set up an e-logbook account and record all procedures you carry out. Stay up to date with relevant courses and examinations.

## Ethical approval

This study did not require ethical approval.

## Sources of funding

This study was not sponsored.

## Author contribution

Miss D Motter contributed to the study concept and writing the paper. Miss F Salimi contributed to interpretation and analysing the paper.

## Registration of research studies

1 Name of the registry:
2 Unique Identifying number or registration ID:
3 Hyperlink to your specific registration (must be publicly accessible and will be checked):

## Guarantor

Miss D Motter takes responsibility for the integrity of the content of the paper.

## Declaration of competing interest

The authors declare that they have no relevant or material financial interests that relate to the research described in this paper.
